# The potential of using non-coding RNAs in forensic science applications

**DOI:** 10.1093/fsr/owad003

**Published:** 2023-07-18

**Authors:** Yawen Li, Zhuoqun Wang, Dikeledi Ishmael, Yehui Lvy

**Affiliations:** School of Basic Medical Sciences, Shanghai University of Medicine and Health Sciences, Shanghai, China; School of Basic Medical Sciences, Shanghai University of Medicine and Health Sciences, Shanghai, China; School of Basic Medical Sciences, Shanghai University of Medicine and Health Sciences, Shanghai, China; School of Basic Medical Sciences, Shanghai University of Medicine and Health Sciences, Shanghai, China; Institute of Wound Prevention and Treatment, Shanghai University of Medicine and Health Sciences, Shanghai, China

**Keywords:** review, circRNA, lncRNA, miRNA, non-coding RNA, forensic science

## Abstract

With the continuous development and integration of molecular biology and forensic science, non-coding RNAs (ncRNAs), especially ncRNAs with regulatory functions such as microRNA, long non-coding RNA, and circular RNA, have recently been actively explored by forensic scholars. In this study, we review the literature on these ncRNAs in various fields of forensic science, including postmortem interval determination, wound age estimation, forensic age assessment, cause of death analysis, and body fluid identification, aiming to evaluate the current research and provide a perspective for future applications.

## Introduction

Non-coding RNAs (ncRNAs) are RNA molecules that do not encode proteins, but instead constitute various classes of housekeeping RNAs. These include the well-known ribosomal RNAs (rRNAs) and transfer RNAs (tRNAs), as well as ncRNAs with regulatory functions that can control gene expression at the transcriptional or post-translational level. These different subclasses of ncRNAs encompass microRNAs (miRNAs), long non-coding RNAs (lncRNAs), and the recently discovered circular RNAs (circRNAs). Many studies have demonstrated that these ncRNAs play critical roles in a wide variety of biological and pathological processes and widely participate in the occurrence and development of various diseases, highlighting why biomedical researchers have strong interest in them [1–3]. The first forensic application was in 2009, when Hanson et al*.* [4] explored the potential of using miRNAs in body fluid identification. Following this, more scholars have been investigating the potential application of ncRNAs in the forensic science field throughout the past decade (Table 1). Because forensic science and molecular biology continue to converge, this review aims to provide an overview of the current knowledge and recent research progress, as well as highlight the potential applications of ncRNAs in forensic science.

**Table 1:** The application of ncRNAs in forensic science.

**Application field**	**Year**	**First Author**	**NcRNA type**	**Characteristic ncRNA markers**	**Tissue/cell type**	**Source**
PMI estimation						
	2013	Odriozola et al. [5]	miRNA	miR-142-5p, miR-541, miR-122	Vitreous fluid	Human
	2015	Corradini et al. [6]	miRNA	miR-106b, miR-96, miR-142-5p, miR-219	Vitreous fluid, blood	Human
	2021	Martínez-Rivera et al. [7]	miRNA	miR-23b-3p, miR-381-3p	Skeletal muscle	Rat
	2015	Sharma et al. [8]	miRNA	miR-2909	Blood, heart and brain	Mouse
	2010	Li et al. [9]	miRNA, rRNA	miR-1-2, 18S rRNA	Myocardium	Rat
	2014	Lv et al. [10]	miRNA	miR-125b, miR-143	Spleen	Rat
	2015	Ma et al. [11]	miRNA	miR-9, miR-125b	Brain	Rat
	2018, 2019	Tu et al. [12, 13]	circRNA, miRNA, rRNA, snRNA	circ-AFF1, LC-Ogdh, LC-LRP6, miR-122, miR-133a, 18S rRNA, U6 snRNA, etc.	Heart, liver and skeletal muscle	Mouse
	2015	Kraus et al. [14]	lncRNA	LUST, IGF2AS, 7SK, etc.	Brain	Human
	2017	Lv et al. [15]	miRNA, rRNA	miR-1, miR-133a, miR-122, 5S rRNA, etc.	Brain, myocardium, liver	Rat, human
Injury time estimation						
	2013	Jin et al. [16]	miRNA	miR-99a, miR-99b, miR-100	Skin	Mouse
	2011	Yang et al. [17]	miRNA	miR-21	Skin	Mouse
	2011	Bertero et al. [18]	miRNA	miR-483-3p	Skin	Mouse, human
	2018	Chang et al. [19]	miRNA	miR-126	Skin	Human
	2015	Fiedler et al. [20]	lncRNA	LINC00323, MIR503HG	HUVECs	Human
	2015	Li et al. [21]	lncRNA	lncRNA 8975-1, AC097662.2	Skin	Human
	2014	Michalik et al. [22]	lncRNA	MALAT1	HUVECs, skin	Human, mouse
	2017	Yang et al. [23]	circRNA, miRNA	circ-Amotl1, miR-17	Skin	Mouse
Age inference						
	2010	Hackl et al. [24]	miRNA	miR-17, miR-19b, miR-20a, miR-106a	Endothelial cells, skin, etc.	Human
	2016	Rubie et al. [25]	miRNA	miR-496	Blood	Human
	2010	Noren et al. [26]	miRNA	miR-103, miR-107, miR-128	Blood	Human
	2016	Li et al. [27]	miRNA	miR-223, miR-130a	Blood	Human
Cause of death determination						
Sudden cardiac death	2009	Terentyev et al. [29]	miRNA	miR-21	Ventricular myocytes	Rat
	2012	Gao et al. [31]	miRNA	miR-122, miR-370	Blood	Human
	2021	Dai et al. [32]	miRNA	miR-24-3p, miR-128-3p	Coronary artery	Human
	2012	Liu et al. [33]	miRNA	miR-106b, miR15b	HUVECs	Human
	2012	Corsten et al. [35]	miRNA	miR-155	Myocardium	Mouse, human
	2021	Zhang et al. [36]	miRNA	miR-155	Myocardium	Human
	2021	Yan et al. [37]	miRNA	miR-3113-5p, miR-223-3p, miR-499a-5p	Heart	Human
	2021	Tian et al. [38]	circRNA	circSLC8A1, circNFIX	Heart	Rat, human
	2013	Courts et al. [39]	miRNA	miR-1, let-7b	Heart, brain	Human
Mechanical asphyxia	2007	Kulshreshtha et al. [42]	miRNA	miR-26, miR-107, miR -210	HT29 cell lines, etc.	Human
	2020, 2021	Han et al. [43, 44]	miRNA	miR-122, miR-3185	Myocardium	Human
	2016	Zeng et al. [45]	miRNA	miR-122	Heart, brain	Human
	2009	Rane et al. [47]	miRNA	miR-199a	Cardiac myocytes	Rat
	2016	Deng et al. [48]	miRNA	miR-103, miR-107	PASMCs	Rat
Poisoning	2015	Sharma et al. [49]	miRNA	miR-2909	Blood, PBMCs	Mouse, human
	2021	Fan et al. [51]	lncRNA	ENSMUST00000137546, etc.	Hippocampus	Mouse
Identification of body fluid						
Body fluid-specific markers	2014	Li et al. [54]	miRNA	miR-214, miR-451a, miR -888, miR-891a	Venous blood, menstrual blood, semen	Human
	2016	Sauer et al. [55]	miRNA	miR-891a-5p, miR-144-3p, miR-203a-3p, miR-124-3p	Venous blood, menstrual blood, saliva, semen, vaginal secretions	Human
	2019	Liu et al. [56]	circRNA	circHBA, circHTN3, circ CYP2B7P1, etc.	Peripheral blood, menstrual blood, saliva, vaginal secretions, semen, urine	Human
	2022	Iroanya et al. [57]	miRNA	miR-451a, miR-10b, miR-205	Blood, semen, saliva	Human
	2022	Bamberg et al. [58]	miRNA	miR-451a, miR-484, miR-214-3p, etc.	Blood, semen, saliva, vaginal secretion, menstrual blood, skin	Human
	2022	Rhodes et al. [59]	miRNA	let-7g, let-7i, miR-200b, miR-320c, miR-10b, miR-891a	Blood, menstrual secretions, feces, urine, saliva, semen, vaginal secretion	Human
Blood (trace) markers	2011	Courts et al. [60]	miRNA	miR-126, miR-150, miR-451	Venous blood, etc.	Human
	2013	Wang et al. [61]	miRNA	miR-16, miR-486, miR-124	Venous blood, menstrual blood, etc.	Human
	2014	Park et al. [62]	miRNA	miR-484, miR-182	Blood, etc.	Human
	2010	Zubakov et al. [63]	miRNA	miR-20a, miR-106a, miR-185, miR-144	Venous blood, menstrual blood, etc.	Human
	2021	Kim et al. [64]	miRNA	miR-208b, miR-1	peripheral blood, pre-/post-cardiac blood	Human
Semen (spot) markers	2013	Wang et al. [61]	miRNA	miR888, miR891a	Semen, etc.	Human
	2014	Park et al. [62]	miRNA	miR-2392, miR-3197	Semen, etc.	Human
	2015	Tong et al. [67]	miRNA	miR-10b, miR-135b	Semen, etc.	Human
	2018	Tian et al. [68]	miRNA	miR-10a, miR-10b, miR-135a, miR-135b, miR-888, miR-891a	Semen	Human
	2019	Wang et al. [69]	piRNA	piR-55521	Semen, etc.	Human
	2019	Hassan et al. [70]	lncRNA	RPS4Y1	Semen, etc.	Human
Markers of menstrual blood and vaginal secretions	2009	Hanson [4]	miRNA	miR-451, miR-124a	Menstrual blood, vaginal secretions, etc.	Human
	2017	Li et al. [71]	miRNA	miR-141-3p, miR-373-3p, miR-497-5p, etc.	Menstrual blood, vaginal secretions, etc.	Human
	2017	Zhang et al. [73]	circRNA	circALAS2, circMMP7	Menstrual blood, etc.	Human
	2019	Wang et al. [74]	piRNA	piR-hsa-27622, piR-hsa-27493, etc.	Menstrual blood, vaginal secretions, etc.	Human
	2022	Wang et al. [75]	miRNA	miR-451a, miR-21-5p	Menstrual blood, peripheral blood	Human

## Application of ncRNAs in estimating the time of death

Accurately inferring the postmortem interval (PMI) is a fundamental problem in forensic pathology. A correct measurement of the PMI can help to delineate the scope of the investigation, identify and exclude suspects, and determine the nature of the case. With the continuous interface of molecular biology and forensic pathology, certain progress has been made with measuring the temporal changes of ncRNA levels to evaluate the PMI. In addition, miRNA measurements may be more reliable in determining the PMI because of their smaller fragment lengths and relatively stable postmortem properties.

### Body fluid organization

Odriozola et al*.* [5] attempted to infer PMI using rhythm gene-related miRNAs by examining the expression levels of 496 miRNAs in seven human vitreous fluid tissues (3 day time deaths and 4 night-time deaths). They selected nine highly abundant miRNAs for further investigation in 34 human samples, using miR-222 as a reference gene. The day–night differences were confirmed for miR-142-5p and miR-541, suggesting that miRNA levels may be related to either the ambient light or the circadian clock at the time of death. Corradini et al*.* [6] further investigated the expression of certain miRNAs in vitreous fluid and blood. Significant differential expression between day and night deaths were found for two miRNAs in the vitreous humour, miR-106b and miR-96, and for two miRNAs in blood, miR-142-5p and miR-219. Despite these findings, the investigation of PMI by measuring miRNA expression in body fluids in a circadian manner is generally lacking. Additionally, it is not known how environmental factors can influence this in a mechanistic way, hampering the application of these measurements in a forensic setting.

### Parenchymal organs

One experiment [7] showed that miR-23b-3p and miR-381-3p are involved in early PMI and may act as EPC1 genes to influence the expression level of certain genes related to the autolysis process. MiR-23b-3p inhibited the activating role of TGIF1 on TGF-β signalling and hypoxia-related genes. Conversely, miR-381-3p promoted hypoxia-generated oxidative stress, apoptosis, and inflammatory inhibition, which are involved in early postmortem autolysis. Another study [8] found that the AATF RNome, consisting of AATF mRNA and miR-2909, exhibits circadian rhythms in the same way in mouse blood and tissues, especially in the heart and brain.

Many studies have found that certain ncRNAs can remain stable in parenchymal organs over a relatively long period of time. These ncRNAs could possibly be used as internal references for determining changes in other RNAs. For example, miR-1-2 in cardiac muscle tissue [9], miR-125b and miR-143 in spleen tissue [10], and miR-9 and miR-125b in brain tissue of rats [11] all showed relatively stable postmortem expression. Similarly, miR-122, miR-133a, and 18S rRNA in heart tissues of rats, LC-Ogdh, circ-AFF1, and miR-122 in liver tissues of rats, and miR-133a, circ-AFF1, and LC-LRP6 in skeletal muscle tissues of rats were found to be relatively stable within 8 days after death [12, 13]. The abovementioned results suggest that miRNAs and circRNAs are more stable as internal controls than other kinds of RNAs for PMI determination. For lncRNAs, LUST, IGF2AS, 7SK, HOXA6as, and NDM29 were selected as stable reference genes among over 90 candidate lncRNAs within a 27-h postmortem period [14]. These findings have been used to establish mathematical models for PMI inference, as validated by Ma et al*.* [11] and Lv et al*.* [15] using human brain tissue samples with different PMIs and causes of death. These data confirm the applicability of the internal reference indicators using a multivariate mathematical model of temperature, PMI, and quantification cycle (Cq) differences of RNA indicators.

For PMI inference, miRNAs have been investigated more frequently, while lncRNAs and circRNAs are still in the initial stages. Although ncRNAs provide a new way of thinking for PMI inference, this analytical method is currently restricted to laboratory situations because of technical limitations and a lack of sufficient data validation in natural environments and human specimens, warranting further research.

## The value of ncRNAs in estimating injury time

Non-coding RNAs are also closely related to the wound healing and scar tissue formation processes and can theoretically be used to infer the time of injury by detecting altered expression of post-injury indicators. Currently, high-throughput sequencing methods can evaluate tissue specimens at the cellular and molecular levels and simultaneously detect multiple injury markers. Among those markers, specific ncRNAs can be screened as potential auxiliary indicators to assess injury time, and the results can be applied to forensic practice.

Using a mouse wound healing model, Jin et al*.* [16] screened 63 differentially expressed miRNAs and found that miR-99a, miR-99b, and miR-100 contribute to wound healing *via* the AKT/mTOR signalling pathway. According to a study by Yang et al*.* [17], miR-21 expression levels were increased 2.8- to 3.9-fold at the wound edge from 1 to 3 days after injury. As reported by Bertero et al*.* [18], miR-483-3p expression peaked at 3 days post-injury and normalized at 6–7 days both in human injury cell healing and mouse injury skin healing models. Recently, Chang et al*.* [19] performed a full-thickness skin incision on the abdomen of 20 healthy volunteers. Real-time quantitative polymerase chain reaction (qPCR) detection and analysis showed that miR-126 expression significantly increased 1–7 days after skin injury and showed an upward trend. These expression levels correlated with the time of injury and at 7 days were four times higher than miR-126 expression levels in normal skin. Therefore, these miRNAs may be useful as candidate indicators for wound time estimation.

Experiments have demonstrated strong hypoxia-dependent activation of two intergenic lncRNAs: LINC00323 and MIR503HG [20]. Li et al*.* [21] screened a set of lncRNAs that had differential expression in scar formation, including lncRNA 8975-1, AC097662.2, and RP11-586K2.1. Related studies demonstrated that lncRNAs LINC00657, TUG1, and metastasis-associated lung adenocarcinoma transcript 1 (MALAT1) are activated by hypoxia in human endothelial cells [22].

Yang et al*.* [23] found that increased Circ-Amotl1 expression can accelerate fibroblast proliferation and migration, and can accelerate wound healing by binding to mitosis-related protein STAT3. Then, circ-Amotl1 promotes Stat3 nuclear translocation and binds to the DNMT3A promoter, thereby enhancing DNMT3A expression and modulating miR-17 function. Both can promote wound healing, and circ-Amotl1 is robustly expressed in wounded tissues.

The above studies demonstrate that certain ncRNAs have a high prospect of be applied to injury time inference because of their post-injury specific expression.

## The value of ncRNAs in age inference

Numerous studies have shown the potential of ncRNAs as novel markers for age inference. Hackl et al*.* [24] identified four senescence-regulated miRNAs (miR-17, miR-19b, miR-20a, and miR-106a), suggesting the potential of miRNAs as a novel marker of human cellular senescence. Recent studies have also confirmed the age-associated expression patterns of miRNAs in various tissues and body fluids [25-27]. A closer mechanistic examination revealed that miR-496 is involved in the regulation of human aging by controlling the mammalian target of rapamycin protein (mTOR) signalling pathway [25]. Noren et al. [26] identified nine miRNAs (miR-103, miR-107, miR-128, miR-130a, miR-155, miR-24, miR-221, miR-496, miR-1538) that were significantly lower in older individuals. Another study suggests that aging may be related to certain miRNA-mediated regulation, including control of FLNB, CDK4, and ZNF274 expression by miR-223, let-7d, and miR-130a, respectively. Thus, this indirectly affects the involvement in MAPK signalling T cell receptor and neurotrophin signalling pathways [27]. Therefore, age-associated miRNAs and their targets have potential utility for inferring age in forensic sciences.

## The value of ncRNAs in determining causes of death

### Sudden cardiac death

Sudden cardiac death (SCD) caused by cardiovascular disease is a recognized priority in forensic testing. With the in-depth research on ncRNAs and the widespread use of high-throughput sequencing technology, a breakthrough in identifying some difficult SCDs has been achieved.

Myocardial infarction (MI) is a common clinical cause of death seen in forensic science cases, while cardiac electrical failure from arrhythmias is the leading cause of SCD in forensic science. There have been reports demonstrating that specific miRNAs play a crucial role in regulating cardiac conduction stability and in the remodeling processes that contribute to the development of arrhythmias [28, 29]. MiR-1 is involved in cardiac electrophysiological activity, primarily by affecting ion channel expression to regulate cardiac excitability and maintain normal cardiac conduction. When miR-1 is overexpressed, it increases Ca^2+^ efflux from the sarcoplasmic reticulum of relaxing cardiomyocytes, leading to an imbalance in intracellular calcium homeostasis and induction of arrhythmias [29]. Decedents of SCD usually suffer from underlying diseases such as coronary artery disease (CAD), cardiomyopathy, and heart valve disease, with CAD being the most common [30]. Several studies have shown that some miRNAs are highly expressed in patients with CAD compared with the healthy population. Gao et al*.* [31] found that miR-122 and miR-370 levels, which are related to lipid metabolism, were significantly increased in CAD patients with hyperlipidaemia. Dai et al*.* [32] showed that miR-24-3p and miR-128-3p expression levels were relatively higher in CAD patients than in controls.

Most patients with SCD have a previous history of MI, which is usually accompanied by changes in the expression patterns of multiple miRNAs. The literature shows [33] that a batch of miRNAs including miR-106b and miR15b appear to be aberrantly expressed in a rat model of MI. From a mechanistic perspective, miR-106b is served as an anti-apoptotic modulator through inhibition of p21 expression and miR-15b displayed anti-angiogenesis activity. These miRNAs played crucial role in the pathogenesis of MI and hold the potential to serve as molecular markers for SCD determination.

A recent review [34] revealed a critical role for miR-155 in various physiological and pathological processes, including inflammation, immunity, and cardiovascular disease. Highly expressed miR-155 can induce cardiac infiltration of macrophages and T lymphocytes in viral myocarditis, exacerbating the myocardial injury and impairing cardiac function [35]. Consequently, genetic alterations in miR-155, such as specific polymorphisms, are associated with cardiac pathology. In a 2021 study [36], researchers screened the entire region of the miR-155 target gene MIR155HG and identified a positive association between rs72014506 and the risk of SCD. Additionally, Yan et al*.* [37] reported that the relative expression levels of miR-3113-5p, miR-223-3p, miR-499a-5p, and miR-133a-3p were significantly increased in SCD samples compared with control samples. Receiver operating curve analysis showed that these four miRNAs could serve as independent diagnostic markers for SCD. Further investigations demonstrated that several miRNA panels consisting of two of the four miRNAs achieved better discriminative power in identifying the cause of SCD. These results suggest that miR-3113-5p, miR-223-3p, miR-499a-5p, and miR-133a-3p are potential candidate biomarkers for diagnosing SCD.

In the studies described above, ncRNAs were shown to be specifically and abnormally expressed in SCD-related diseases. The potential pathogenic mechanisms involve the detection of myocardial ion channel-related ncRNA expression, which could be a breakthrough in the forensic diagnosis of SCD. Tian et al*.* [38] investigated the expression of circSLC8A1 and circNFIX in myocardium in different types of commonly used ischemic heart disease (IHD) models, and further validated their expression in forensic autopsy cases. They found that expression level of circSLC8A1 was increased, while circNFIX levels were elevated in the early phase of ischemia and subsequently decreased in IHD samples. Further analysis showed that circSLC8A1 exhibited high sensitivity and specificity for MI and was positively correlated with creatine kinase MB levels in pericardial fluid. Decreased circNFIX may indicate ischemic myocardial injury and be negatively correlated with coronary stenosis grade. The combination of circSLC8A1 and circNFIX showed better performance in identifying IHD-related SCDs. The time-dependent expression patterns of these two circRNAs suggested that they could serve as auxiliary diagnostic markers for acute IHD-induced SCD in forensic work. Courts et al*.* [39] found that cardiac-specific miR-1 is significantly increased in sudden infant death syndrome and could be used as molecular markers to assist with determining the cause of death in infants. With further investigation, other ncRNAs, for example certain circRNAs and mechanisms of action related to SCD may be discovered, providing new directions and ideas for identifying SCD [40].

### Mechanical asphyxia

The diagnosis of mechanical asphyxiation death mainly uses postmortem marks, crime scene analysis, and case information. However, signs of suffocation in the body, such as facial cyanosis, visceral congestion, Tardieu spots, and rose teeth, are all non-specific signs. In some cases, where the circumstances of the case are unknown, the crime scene is destroyed, or the autopsy examination lacks the damage traces of mechanical external force that can lead to suffocation, it is often difficult to determine whether the death was from mechanical suffocation. However, ncRNAs are more suitable as molecular markers for inferring the cause of mechanical asphyxiation death because of their own small fragments and relatively slow postmortem degradation.

Hypoxic conditions can induce the secretion of surfactant protein A (SP-A). The duration and intensity of hypoxia from asphyxia are longer and more intense than other causes of death. Increased SP-A levels in cases of mechanical asphyxia suggest that hypoxia is a prominent feature of death in such cases [41]. Kulshreshtha et al*.* [42] first reported that hypoxia can affect miRNA expression. Some miRNAs were aberrantly expressed in many specimens from humans that died by mechanical asphyxia, including death by hanging and drowning. Han et al*.* [43] analysed 156 human heart tissue samples and developed a molecular prediction model using multiple indicators (DUSP1, KCNJ2, miR-122 and miR-3185) to determine mechanical asphyxia death, which could be used as a potential molecular marker. Another article from this group [44] suggests that the miR-3185/CYP4A11 axis is associated with mechanical asphyxia death, which provides new insights into death investigations in these cases. Zeng et al*.* [45] found that in the brain and heart tissues of those who died by mechanical asphyxia, miR-122 expression was significantly decreased compared with craniocerebral injury and haemorrhagic shock. Cecchi et al. [46] showed that hypoxia-inducible factor 1-α (HIF-1α) exhibits high expression in hypoxic lung tissues. HIF1α is targeted by multiple miRNAs, making them potential hypoxia biomarkers. Rane et al*.* [47] found that miR-199a, which targets and inhibits HIF-1α, is a critical inducer of hypoxia-triggered regulatory pathways, and the decreased miR-199 could support hypoxia-induced repression of pro-apoptotic genes caspase-3, caspase-6, caspase-9, and caspase-12, as well as FasL, AIF, and Bnip1. Deng et al*.* [48] showed that hypoxia reduces miR-103/107 levels in pulmonary artery smooth muscle cells, which leads to increased expression of HIF-1β. According to the above pathways, miRNAs play crucial roles in hypoxia from asphyxia and can be of great help in determining the cause of death.

### Poisoning

MiR-2909 can significantly increase T-cell populations and Th1-positive cytokines, thereby regulating nonspecific immunity. According to Sharma et al*.* [49], miR-2909 expression levels were elevated in mice with sodium arsenite poisoning. This study suggests that in cases of chronic poisoning by toxicants, miRNA levels change with a specific pattern and can be used as co-biological factors for toxicant analysis and inference of the cause of death. Another review [50] showed that miR-21 can alter Th2 and Th1 homeostasis by regulating IL-12 expression, and the increased amount of miR-146a inhibits Treg-mediated responses while enhancing Th1 responses, suggesting that certain miRNAs could be biomarkers for determining the cause of death in allergic reactions. Fan et al*.* [51] found that abnormal expression of lncRNAs was associated with exposure to glyphosate in the perinatal period.

These experiments and studies demonstrate that ncRNAs play various regulatory roles in the development of various diseases. They are involved in the regulation of different processes, including electrophysiological activity, inflammation, and immune responses. In contrast, abnormal expression patterns of ncRNAs have been observed in many specimens of different causes of death. Some ncRNAs are stably expressed and do not change with rhythm, allowing them to be used as internal references to observe the expression patterns of other ncRNAs. Yet, certain ncRNAs are only expressed in specific causes of death. Some have rhythmic changes, which can help forensic pathologists investigate the specific cause of death and infer the time of death, suggesting that ncRNAs have broad application prospects in forensic science.

## The value of ncRNAs in the identification of body fluid

### Body fluid-specific markers

Identifying and determining the source of body fluids at crime scenes is of critical importance in forensic practice. Early studies on the forensic identification of body fluids mainly used analytical methods, such as immunological techniques or biochemical assays but were inevitably restricted in practical application by limitations such as sample consumption, the labour-intensive and time-consuming nature of the work, and varying degrees of sensitivity and specificity [52]. One alternative to traditional methods was mRNA analysis for fluid body identification because of their tissue specificity. However, it can be easily affected by the external environment and degraded by internal and external mRNA enzymes, making it not suitable for detecting corrupt samples [53]. Certain ncRNAs are characterized by specific expression patterns in various tissues and cells and are more stable and not easily degraded in different environments. Li et al*.* [54] established a coextraction and co-analysis method for miRNA and DNA somatic fluid identification using linear reverse transcription primers and obtained four miRNA markers and DNA short tandem repeat (STR) profiles from the same sample. Thereafter, Sauer et al*.* [55] screened four miRNAs to distinguish venous blood, menstrual blood, saliva, semen, and vaginal secretions, and Liu et al*.* [56] identified 14 circRNA-specific expression patterns in five body fluids: menstrual blood, saliva, vaginal secretions, semen, and urine. This indicated the biomarker potentials of ncRNAs for body fluid identification. Recently, Iroanya et al*.* [57] ascertained the stability of miRNA markers miR-451a, miR-10b, and miR-205 in blood, semen, and saliva exposed to different environmental conditions. These markers are stable in different environments (outdoor, indoor, fridge, and freezer), showing that these biomarkers had forensic utility for body fluid identification. Bamberg et al*.* [58] simultaneously analysed mRNA and miRNA markers, reporting that miRNA markers were more advantageous for examining degraded samples. Another study [59] used an miRNA panel to classify seven forensically relevant body fluids, with their data suggesting that miR-200b, miR-320c, miR-10b, and miR-891a, when normalized to let-7g and let-7i, can consistently and robustly be used to classify blood, faeces, and urine. In addition, several signature RNAs exist and can help to determine the identity of a person. This may be useful in profiling RNAs of unidentified human remains, which can then be compared with potential relatives. Robust matching between the two datasets can help to identify missing individuals. Therefore, it is imperative to conduct RNA profiling of all unidentified human remains and compare them with potential relatives or the profiles of a generalized population. In addition, this approach may also help identify the survivors of trauma, including disaster victims, who are comatose or disabled and cannot talk.

### Blood (trace) markers

Courts and Madea [60] performed a global screening of c.800 miRNAs in forensic blood and saliva samples by microarray analysis. By bioinformatics processing, they proposed a miRNA assay consisting of three differentially expressed miRNAs for identifying blood (miR-126, miR-150, and miR-451). Through qPCR array screening and subsequent TaqMan-qPCR validation, Wang et al*.* [61] ascertained venous blood-specific miR-486 as a more sensitive biomarker from seven miRNAs with potential humoral specificity. Park et al*.* [62] suggested that miR-484 and miR-182 levels could also be used to identify venous blood. Among 718 human miRNAs, Zubakov et al*.* [63] used the gene microarray method to determine that miR-144 and miR-185 are specifically expressed in blood. Some studies [64] confirmed that cardiac-specific miR-208b and myocardial-specific miR-1 in the blood are expressed at different levels in the pre- and post-cordial regions. Therefore, the characteristics of target miRNAs, such as tissue specificity, should be considered in forensic applications, and sampling sites for miRNAs should be provided. As for other ncRNAs, Salzman et al*.* [65] found that circRNA expression is tissue cell-specific, with different circRNA expression profiles in different cell types. Some circRNAs are also relatively conserved among species, for example, 69 circRNAs in mouse testis are also present in human cells [66]. Therefore, circRNAs show great potential in forensic body fluid identification. Although preliminary research [56] has been conducted, additional work is still needed to identify suitable circRNA markers and stable reference genes for bloodstain identification.

### Semen (spot) markers

Several studies have demonstrated [61, 62, 67] that a variety of miRNAs have significantly high expression in semen, such as miR-888, miR-891a, miR-2392, miR-3197, miR-10b and miR-135b, and can be used as markers for identifying this body fluid. Notably, Tian et al*.* [68] evaluated the expression levels of a set of semen-specific miRNA markers (miR-10a, miR-10b, miR-135a, miR-135b, miR-888, and miR-891a) using real-time quantitative PCR and specific fluorescently labelled TaqMan probes. Their data revealed that compared with standard semen samples, samples from infertile individuals showed a significant decrease in expression of these miRNA markers. Additionally, Wang et al. [69] found that piRNA (Piwi interacting RNA) piR-55521 was specifically expressed in semen. While Hassan et al. [70] found long non-coding XIST was detected in female body fluid and RPS4Y1 was specifically detected in semen and male blood. Taken together, the tissue-specific ncRNA expression can distinguish semen from venous blood, saliva, menstrual blood, and vaginal secretions, which is a novel finding for sex identification of body fluids.

### Markers of menstrual blood and vaginal secretions

Hanson et al*.* [4] applied miRNAs for the first time to identify forensically relevant body fluids and screened three miRNAs (miR-195, miR-124a, and miR-451 for vaginal secretions and menstrual blood). A study by Li et al*.* [71] also screened five menstrual blood-specific miRNAs, namely miR-141-3p, miR-373-3p, miR-497-5p, miR-143-5p, and miR-136-5p. Song et al*.* [72] detected circRNAs in five forensically relevant body fluids using microarray technology and found that the expression profiles of menstrual blood and vaginal secretions showed similar characteristics. In contrast, the expression profiles of saliva, semen, and venous blood were relatively tissue specific. Zhang et al*.* [73] suggested that detecting circRNAs derived from the same gene at the same time as mRNA typing could improve the stability and sensitivity of detecting menstrual blood. The analysis performed by Wang et al*.* [74] confirmed the potential of three piRNAs (piR-hsa-27 622, piR-hsa-1207, and piR-hsa-27 493) for distinguishing venous blood from menstrual blood and two piRNAs (piR-hsa-27 493 and piR-hsa-26 591) for distinguishing saliva from vaginal secretions. A recent study [75] used the ratio of miR-451a to miR-21-5p to distinguish menstrual blood from peripheral blood. These findings expanded the number of potential piRNA biomarkers and demonstrated that the expression profile of piRNAs can provide valuable information for distinguishing forensically relevant biological samples.

## Conclusion and outlook

Compared with mRNAs and other ncRNAs, miRNA expression is more stable and tissue specific. Their utility has been more extensively investigated for determining the time of death, cause of death, and identification of body fluid sources. However, there is no unified standard for the quantitative detection of miRNAs, limiting its application in forensic practice. LncRNAs have relatively few applications in forensic science, mainly for time of death inference and cause of death analysis, and the relevant research is still in its infancy. CircRNAs have significant advantages in detecting old and degraded specimens because of their stability and tissue-specific expression. This class of RNAs can be used as a new biomarker in forensic identification, but further research to gather empirical evidence is needed. Collectively, with the development of molecular biology cross-discipline methods, ncRNAs have broad application prospects in forensic science. They can provide new research directions for the time of death inference, cause of death analysis, body fluid source analysis, and age inference, and deserve the key attention of related scholars.

## Authors' contributions

Yawen Li and Zhuoqun Wang contribute equally to this work. Yehui Lvy, Yawen Li and Zhuoqun Wang designed the framework of the review and drafted the manuscript. Ishmael Dikeledi conducted initial proofreading. All the authors contributed to the final text and approved it.

## Compliance with ethical standards

This article does not contain any studies with human participants or animals.

## Disclosure statement

The authors declare no conflicts of interest.

## Funding

This work was supported by the Shanghai Sailing Plan [21YF1418800] and research scholarship from the International Committee of the Red Cross.
